# Blue and Red LED Illumination Improves Growth and Bioactive Compounds Contents in Acyanic and Cyanic *Ocimum basilicum* L. Microgreens

**DOI:** 10.3390/molecules22122111

**Published:** 2017-11-30

**Authors:** Andrei Lobiuc, Viorica Vasilache, Olga Pintilie, Toma Stoleru, Marian Burducea, Mircea Oroian, Maria-Magdalena Zamfirache

**Affiliations:** 1 Faculty of Food Engineering, Stefan Cel Mare University, Universitatii Street 13, 720229 Suceava, Romania; andrei.lobiuc@fia.usv.ro (A.L.); m.oroian@fia.usv.ro (M.O.); 2 CERNESIM Research Center, Alexandru Ioan Cuza University, Carol I Boulevard 20A, 700506 Iasi, Romania; 3 Interdisciplinary Research Department-Field Science, Alexandru Ioan Cuza University, Lascar Catargi 54, 700107 Iasi, Romania; 4 Faculty of Chemistry, Alexandru Ioan Cuza University, Carol I Boulevard 20A, 700506 Iasi, Romania; olgapintilie@yahoo.com; 5 Faculty of Biology, Alexandru Ioan Cuza University, Carol I Boulevard 20A, 700506 Iasi, Romania; toma.stoleru@gmail.com (T.S.); marian.burducea@yahoo.com (M.B.); magda_zamfirache@yahoo.com (M.-M.Z.)

**Keywords:** basil, anthocyanins, light emitting diodes, chlorophyll fluorescence, phenolic acids

## Abstract

Microgreens are an excellent source of health-maintaining compounds, and the accumulation of these compounds in plant tissues may be stimulated by exogenous stimuli. While light quality effects on green basil microgreens are known, the present paper aims at improving the quality of acyanic (green) and cyanic (red) basil microgreens with different ratios of LED blue and red illumination. Growth, assimilatory and anthocyanin pigments, chlorophyll fluorescence, total phenolic, flavonoids, selected phenolic acid contents and antioxidant activity were assessed in microgreens grown for 17 days. Growth of microgreens was enhanced with predominantly blue illumination, larger cotyledon area and higher fresh mass. The same treatment elevated chlorophyll a and anthocyanin pigments contents. Colored light treatments decreased chlorophyll fluorescence ΦPSII values significantly in the green cultivar. Stimulation of phenolic synthesis and free radical scavenging activity were improved by predominantly red light in the green cultivar (up to 1.87 fold) and by predominantly blue light in the red cultivar (up to 1.73 fold). Rosmarinic and gallic acid synthesis was higher (up to 15- and 4-fold, respectively, compared to white treatment) in predominantly blue illumination. Red and blue LED ratios can be tailored to induce superior growth and phenolic contents in both red and green basil microgreens, as a convenient tool for producing higher quality foods.

## 1. Introduction

Microgreens, the plantlet stage of various species, have been consumed for a long time, especially in the Asian countries, but also in Western countries in the last decades, for the various flavors, textures and tastes they can impart to food [1,2]. Usually produced in ecological setups, microgreens are consumed raw, retaining all the chemical constituents unaltered and are considered functional foods, providing, besides nutrient intake, positive health related effects [3].

In plant cultivation, various factors may be used in controlled conditions to induce physiological changes in the plant [4]. Plants respond to these stressors by activating a series of mechanisms, similar to responses to pathogenic or environmental stimuli, affecting plant metabolism and increasing synthesis of phytochemicals [5]. This effect is highest in young plants, which have to adapt quickly to fluctuating environmental factors to ensure survival. Among different types of factors, physical ones such as temperature and light influence plant metabolism in a complex manner, since they may alter the expression of competent genes [6].

Light quality and quantity directly influences plant growth and chemical composition; therefore, it can be used as a convenient and highly modifiable factor to obtain vegetal material with tailored composition to specific applications. As chlorophyll pigments absorb mainly in the red (663 nm and 642 nm) and blue (430 nm and 453 nm) regions of light, these wavelengths are the main ones that influence plant growth [7,8]. Red light is sensed in plants by phytochrome receptors (PhyA, PhyB, etc.), presenting two interconvertible Pr and Pfr forms, and it generates responses related to germination, stem elongation, leaf expansion, flowering induction, etc. [9]. Blue light is sensed by cryptochromes and phototropins, and regulates processes such as de-etiolation, phototropism, chloroplast movement, endogenous rhythms, growth of roots, light-induced stomata opening, redox balance, the level of cyclic nucleotides, etc. [10,11]

From a biotechnological point of view, cultivating plants under red light supplementation may lead to increased biomass, higher phenolic contents, activation of antioxidant system, increased luteolin and sinigrin concentrations, etc. At the same time, blue light may lead to increased chlorophyll synthesis, phenolic contents and vitamin C contents in various species, as reviewed in Olle and Viršilė [12]. However, better results are obtained when red and blue light are combined compared to monochromatic treatments, which may offer significant plant yield. Red light matches the absorption maxima of chlorophylls, while blue light induces stomata opening and, thus, allows for better CO_2_ fixation. The ratio of red to blue light must be carefully selected, as blue light may induce thresholds over which yield decreases may be registered [13,14].

As already noted [14,15], the global requirements of increased food production, areas with limited arable surfaces or daylight, particular climatic conditions, made Controlled Environment Agriculture (CEA) an attractive alternative for producers. Using LED sources with defined wavelength in such environments presents a series of benefits such as maximized growth, control of morphology, optimized flavor and pigmentation, and increased accumulation of bioactive compounds.

*Ocimum basilicum* L. is a species widely used in food preparations, containing high amounts of phenolic constituents with antioxidant activity, substances that are able to delay or inhibit the oxidation of lipids or other molecules by interfering with oxidative chain reactions, to neutralize free radicals, to quench single and triplet oxygen species and to decompose peroxides [16]. Many cultivars exist, of which many are red pigmented, with differences in chemical composition [17]. Basil microgreens were proven to contain high amounts of phenolic contents and consumer acceptability [18].

The effects of LED illumination in microgreen cultivation has been investigated in several species, such as *Brassica oleracea*, *B. juncea*, *B. rapa* [19,20], pea, broccoli, mustard, borage, amaranth, kale, beet, parsley [21], *Valerianella locusta* [22], buckwheat [23], *Perilla frutescens* [24], etc. In basil microgreens, research on light quality regarded the effect of red light supplementation on total phenolic content and antioxidant activity [21]; red, blue and far-red supplementation on antioxidant activity [25]; ultraviolet A (UV-A) supplementation on growth, phenolic, anthocyanin, ascorbate and tocopherol synthesis [24]; and blue light dosage on growth, ascorbate, total phenolic, anthocyanin, flavonoid contents and 2,2-diphenyl-1-picrylhydrazyl (DPPH) antioxidant activity [25]. However, all respective studies tested only a green basil cultivar, “Sweet Genovese”.

The present paper aims to test the following hypotheses: (a) growth of basil microgreens may be influenced by different red:blue light ratios; and (b) depending on the presence of anthocyanins, the metabolic pathways of basil may be differently influenced by light quality, resulting in different quantitative chemical composition. For these purposes, two basil cultivars are employed. One was represented by the same green cultivar “Sweet Genovese” as in other reports to establish a line of comparison and a red leaved cultivar “Red Rubin”, for assessing the influence of the presence of anthocyanins on the effect of light quality on basil chemical composition.

## 2. Results

**Figure 1:**
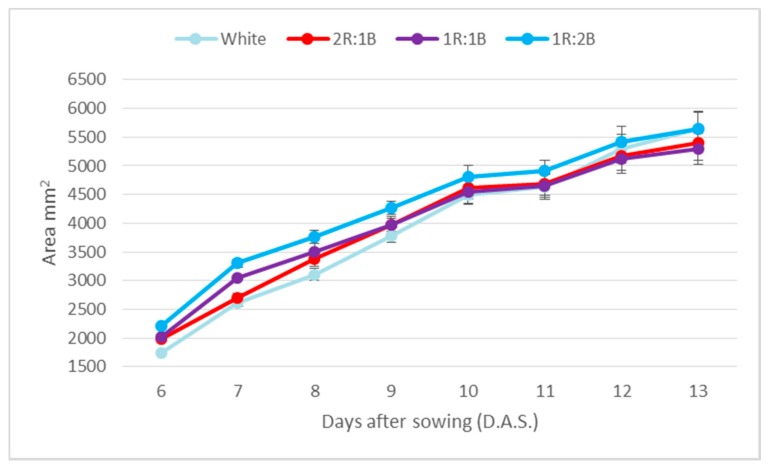
Total cotyledon area of green basil cultivar.

The light treatments did not significantly influence the total leaf area of the green basil cultivar (Figure 1), however the highest area was constantly recorded for the 1R:2B treatment during the eight measurement days. All other treatments had similar values, especially over Days 10–13 of measurement. The results may be related to the composition of light because, although the first point of measurement (six days after sowing) shows differences between treatments, it has to be considered that the plantlets had already been under the influence of light from Day 3 after sowing.

Plant fresh and dry mass registered highest values for the 1R:2B light treatment, compared to white light, with a 1.29-fold increase. The 2R:1B and 1R:1B treatments recorded lower values than white light (Table 1). Although a correlation between increases in mass and ones in leaf areas may seem plausible, such a correlation was not established in the present study, as mass assessments were performed only on the final day of the experiment.

Chlorophyll a contents were different, but not significantly, in the two cultivars, with more pigments in the green cultivar compared to the red one, while chlorophyll b and carotenoids content were similar in both cultivars (Table 2). The higher ratios of blue wavelength in the light treatments led to increases in both chlorophyll a and chlorophyll b, for both cultivars, while carotenoids content remained generally the same.

**Figure 2:**
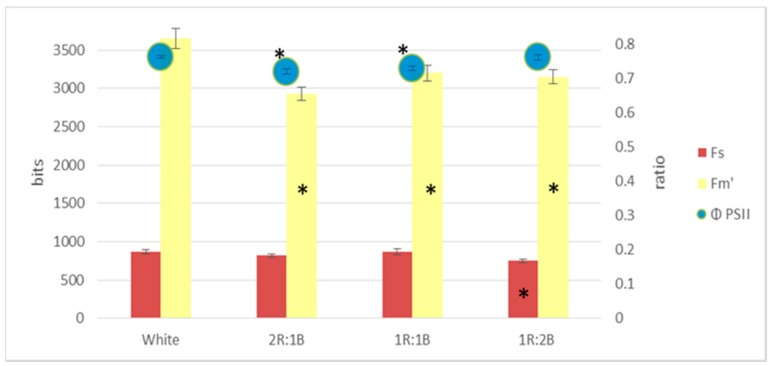
Chlorophyll fluorescence parameters in green basil cultivar (* represents significant differences from white light treatment for *p* < 0.05).

**Figure 3:**
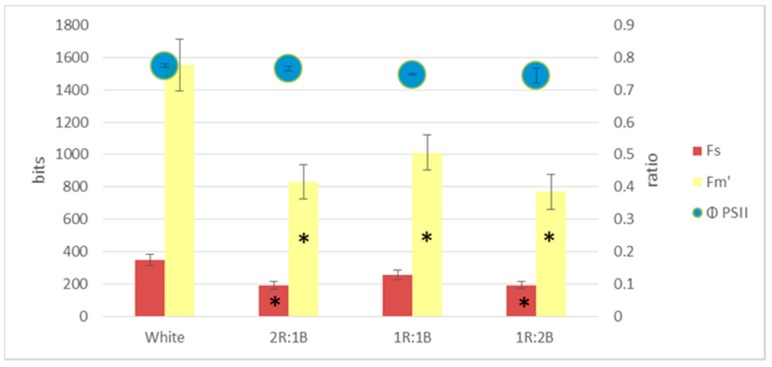
Chlorophyll fluorescence parameters in red basil cultivar (* represents significant differences from white light treatment for *p* < 0.05).

Regarding the fluorescence indices, compared to white light, all other treatments led to significant decreases of the maximum fluorescence (Fm’), with larger differences for the red cultivar (Figure 2 and Figure 3). The 1R:2B ratio also induced lower Fs values for both green and red cultivars. However, the quantum yield of photosystem II (ФPSII) was significantly different only for the 2R:1B and 1R:1B treatments, in the green cultivar, as a result of lowered values only for Fm’ but not for Fs.

**Figure 4:**
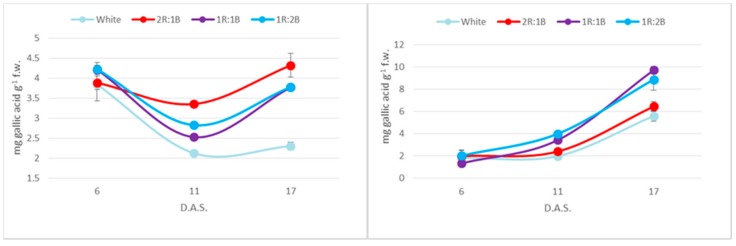
Total phenolic contents of: green basil cultivar (**Left**); and red basil cultivar (**Right**).

Phenolic contents were overall higher in the red cultivar, presumably as a result of anthocyanin presence (Figure 4). Considering the light treatments, the two cultivars behaved differently, as the maximum phenolic accumulation was recorded for 2R:1B treatment in the green cultivar and for the 1R:1B and 1R:2B ratios in the red one. The differences were not statistically significant (*p* < 0.05) for values at six days after sowing. At 11 and 17 days after sowing, statistically different values compared to white light were registered for all colored treatments for the green cultivar and for 1R:1B and 1R:2B for the red one. The increases compared to the white treatments after 17 days were 1.87-fold and 1.63-fold in the green cultivar, and 1.73-fold, 1.58-fold and 1.15-fold in the red one. Another difference between cultivars related to the evolution of phenolic contents over time, as in the green cultivar there is a decrease at 11 days after experiment initiation, followed by an increase at 17 days. Over the same period, the red cultivar recorded a continuous increase of values.

**Figure 5:**
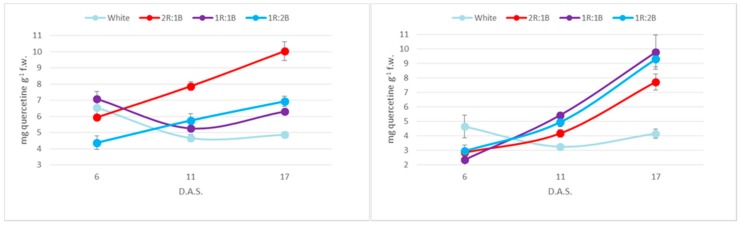
Flavonoid contents of: green basil cultivar (**Left**); and red basil cultivar (**Right**).

A similar trend was recorded for flavonoid contents, where the 2R:1B induced the highest accumulation for the green cultivar (2.06-fold increase compared to white light) and the 1R:1B (2.35-fold) and 1R:2B (2.23-fold) in the red one (Figure 5). Statistically different values compared to white light were recorded at 11 days after sowing for 2R:1B for the green cultivar and for 1R:1B and 1R:2B for the red one. At 17 days after sowing, differences from white light were statistically different for 2R:1B and 1R:2B in green basil and for all colored treatments in the red basil. Overall, the two cultivars had similar amounts of flavonoids.

**Figure 6:**
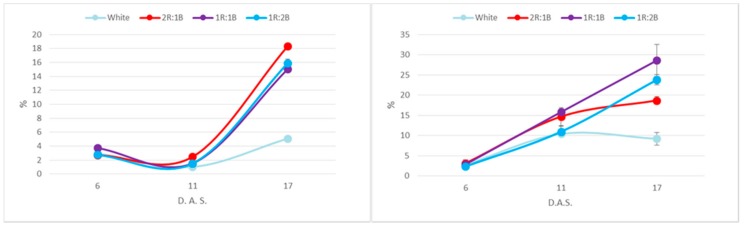
Free radical scavenging capacity of: green basil cultivar (**Left**); and red basil cultivar (**Right**).

Free radical scavenging capacity followed closely the phenolic accumulation in the microgreens, with higher capacity at 2/3 red in the green cultivar and at 1R:1B and 1R:2B in the red one (Figure 6). In addition, the antioxidant capacity presented the same inflexion at 11 days in the green cultivar as did the phenolic contents. Significant statistical differences compared to the white light treatment were observed at 11 days after sowing in the green cultivar for the 2R:1B treatment. At 17 days after sowing, significant differences were observed between white light and all colored treatments in the green cultivar and between white light and 1R:1B and 1R:2B in the red cultivar. The differences between cultivars observed in phenolic contents were also noted for the antioxidant capacity, with much higher values for the red cultivar.

Anthocyanin contents were higher for all treatments compared to white light, however 2R:1B had the highest accumulation (Table 3). For individual phenolic acids of the red cultivar, the light treatments induced effects consistent with those seen for total phenolics and flavonoids. Both caffeic and rosmarinic acid synthesis was stimulated most by the 1R:2B treatment, followed by the 1R:1B, 2R:1B and white light treatments (Table 4). The increase by 1R:2B light in rosmarinic acid was 15-fold compared to white light, while the increase in caffeic acid was four-fold.

## 3. Discussion

Light quantity and quality are essential factors that influence plant development and composition, as light is both an energy source and a regulating signal [26]. It is known that red light is used most efficiently in photosynthesis, but other wavelengths are necessary to maximize growth and plant physiology. For example, blue light, among others, regulates stomatal opening, therefore improving access to CO_2_ and transpiration and is also required to prevent “red light syndrome”, exhibited through suboptimal morphology and modified gene expression and biochemistry [15].

Our results show that blue light promoted cotyledon development and biomass accumulation in the green cultivar. Such effects can be attributed to a better CO_2_ fixation as a result of blue light controlled stomatal opening [13] or to higher nitrogen content in leaves exposed to blue light following the enhancement of nitrate reductase activity [27]. Blue light is known to also influence several enzymes of the carbohydrate dissimilation pathways, such as glucose-6-phosphate dehydrogenase, nicotinamide adenine dinucleotide (NAD)-dependent glyceraldehyde-3-phosphate dehydrogenase, pyruvate kinase, as well as some tricarboxylic acid (TCA) cycle enzymes such as isocitrate dehydrogenase, succinate dehydrogenase or fumarase [28,29]. Higher proportions of blue light are associated with development of “sun type” leaves, as observed by Macedo et al. [11], where, compared to red and white treatments, *Alternathera brasiliana* plants grown with blue light had higher specific leaf mass, number of leaves, and leaf and palisade layer thickness. Such response is targeted at increasing available surface for light harvesting and its penetration to the level of chloroplasts, and is probably mediated by both cryptochromes and phototropins. However, the response is believed to be species specific, as, in other species such as *Lycopersicon*, highest mass, carbohydrates contents and activities of sucrose metabolism enzymes were achieved with higher red ratios (3R:1B) supplemented with blue light, while red treatment alone recorded lower values [30]. Similarly, higher biomass was achieved with 90R:10B or 70R:30B in *Valerianella locusta* [22] or 10R:1B in coriander [31]. In lettuce, the highest mass and leaf area were obtained by white light addition to 1R:1B treatments [8]. In “Sweet Genovese” basil, highest leaf areas were recorded for 100% red and for 33% blue and lower values for 8–25% blue, but the differences were not significant [25].

Chlorophyll synthesis in the present experiment was increased in both cultivars by higher blue light concentrations and this is an expected result, as blue light is considered a driving factor for chlorophyll production. Blue light improves gene expression such as *MgCH*, *GluTR* and *FeCH*, involved in chlorophyll synthesis, while red light at high fluence leads to a reduction in 5-aminolevulinic acid, a tetrapyrrole precursor required for chlorophyll synthesis [32]. Blue light also regulates some of the enzymes in the chlorophyll synthesis pathway, such as phosphoenol pyruvate (PEP)-kinase [33], dioxovalerate (DOVA)-dehydrogenase, DOVA-transaminase, aminolevulinic acid (ALA)-synthase and ALA-dehydratase [28]. In two cultivars of rice, Chen et al. [27] obtained similar chlorophyll a contents among red, blue, green and 1R:1B treatments in one cultivar, and an increase in the 1R:1B treatment in the other. In Chinese cabbage, red:blue 6:1 and blue light increased chlorophyll concentrations significantly, while red light decreased chlorophyll accumulation as a result of reductions in chlorophyll precursors ALA, Proto IX, Mg-Proto IX and protochlorophyllide [32]. In rice seedlings, blue light led to chlorophyll a levels similar to those of white light illuminated plants, as did with levels of Proto IX and Mg-Proto IX, while red light decreased the same precursors [34]. In the same paper, blue light upregulated the transcription of *HO2*, encoding heme oxygenase and of *CHLD*, encoding the D subunit of Mg-chelatase, while red light increased the transcription levels of *PPO1*, encoding protoporphyrin oxidase, underlining the importance of these genes in plant adaptation to altered light environments. Carotenoids levels in our study were not significantly altered by the light treatments, as it is known that carotenoid accumulation maxima may occur at both 440 nm and 640 nm [35]. Similarly, no difference between red, blue or 1R:1B treatments were observed in two rice cultivars [27]. To date, there are few studies regarding the effect of different wavelength on carotenoid production, an example being the one of Fu et al. [36], where it was established that red light increases carotenoid production only up to 128 μmol m^−2^ s^−1^ and addition of blue light improves carotenogenesis. Carotenogenesis is considered to be regulated together with chlorophyll synthesis under the perception of light by both phytochromes and cryptochromes [37].

The effective quantum yield of photosystem II, ΦPSII, is related to the light adapted state of a leaf and, mathematically, it is a product of the maximum photosystem II (PSII) efficiency (Φp or Fv’/Fm’) and the PSII efficiency factor (qP or Fq’/Fv’) [38]. The first parameter describes the maximum efficiency of a leaf under a given photon flux density and may be used to describe non-photochemical changes that occur under light condition. The second one indicates the amount of active PSII reaction centers that are in an oxidized state and may be used to drive electrons through the system, representing the ability of PSII to maintain reaction centers open or, otherwise stated, the photochemical quenching ability. The basil microgreens in our experiment recorded lower ΦPSII values in all coloured treatments compared to white light, indicating a downregulation of PSII efficiency. This may be the result of changes in non-photochemical quenching, possible reasons, among others being modifications of nicotinamide adenine dinucleotide phosphate (NADP) and adenosine triphosphate (ATP) use leading to thylakoid lumen acidification and carotenoid interconversion or inadequate CO_2_ availability as a result of stomatal regulation [39]. However, considering that blue light promotes stomatal opening and CO_2_ fixation [40], the decreases of ΦPSII appear to be less likely owed to changes in CO_2_ availability. An evaluation of carotenoid interconversion to non-photochemcial quenching (qE component) was not available, as it requires individual quantification of violaxanthin and zeaxanthin rather than of the total amount of carotenoids, which may not change under photooxidation [41]. A possible mechanism might be a decrease in the qT component of non-photochemical quenching, which consists in migration of light harvesting centers from PSII to photosystem I (PSI) to provide correct balancing of energy between the photosystems in low light [42], as in the current experiment. Regarding photochemical quenching, reductions of the proportion of active PSII reaction centers was proposed a possible cause of lower PSII efficiency, as it is known that blue light induces PSII alterations by inactivating the oxygen-evolving complex and, subsequently, the reaction centers [43]. Similar effects to the present ones were recorded in *Cordyline australis* and *Sinningia speciosa*, where red, 1R:1B and blue light decreased ΦPSII and qP values compared to white light, but not in *Ficus benjamina*, where ΦPSII was highest under blue light [44]. It appears that other factors, including the species, influences the response of PSII to the light quality, as in *Mesembryanthemum crystallinum*, increased blue doses led to increased carotenoid synthesis, qP and non-photochemical quenching (NPQ) [45]. Moreover, the response may be cultivar-dependent, as, in our experiment, the green cultivar recorded significant decreases of ΦPSII for two treatments, while no significant changes were observed in the red cultivar. Similar behavior was recorded for *Phalaenopsis*, where a green cultivar was more sensitive to colored treatments than a red one and decreases of qL, the oxidation state of PSII, occurred [46]. A possible explanation may be that the presence of anthocyanins absorbs part of the high energy, stress inducing, blue light [47]. In *Lactuca*, it was proved that red over green tissues sustained less photoinhibition and superoxide anion generation by light. Consequently, red tissues had higher ΦPSII and qP and lower qNP values as a result of better absorption of light by anthocyanins [48]. However, the decreases of ΦPSII values in our experiment in colored treatments compared to white light indicate a stressful condition of microgreens, especially considering that these values are normally around 0.84 in healthy organisms [42].

The effect of the light quality on the synthesis of phenolic substances in the present study was different, depending on the cultivar. The green cultivar was most stimulated by higher proportion of red light, while the red cultivar, by higher ratios of blue light. There is long standing evidence for the photoinduction of phenolics synthesis by light, especially by the blue region of it [49]. At least a key enzyme in the synthesis of phenolics is influenced by light, such as phenylalanine ammonia lyase (PAL) and a mechanism of control of such enzymes is the accumulation of their products, hydroxycinnamic acids [50,51]. More specifically, phenolic synthesis control is done by the transformation of hydroxycinnamic acids, from the *trans* form, strong inhibitors of PAL, to the *cis* form, less inhibitory, by blue light [52]. Particularly, the production of flavones and flavonols is regulated by UV and blue light through chalcone synthase (CHS), the first key enzyme in this pathway, by inducing CHS mRNA accumulation in exposed tissues [53]. Similarly, *smTAT*, a gene encoding tyrosine aminotransferase, involved in rosmarinic acid synthesis, was found to be light stimulated by UV wavelengths [54]. At the same time, UV receptors in plants, represented by cryptochromes and phototropins, are also blue light sensors [55], and their involvement in accumulation of phenolic acids under wavelengths close to UV ones may be presumed [56]. The differences between the response of cultivars to blue and red light might be explained by different regulatory mechanisms of PAL in green and red tissues. The involvement of phytochrome in green tissues but not in red ones was proposed, together with a possible coregulation of PAL and anthocyanin accumulation under the effect of light [51]. It was also stated that regulation of gene expression by light may be different between green and red tissues, even for the same species, such as *Perilla* [57].

The higher amount of rosmarinic and caffeic acids under blue light may be interpreted taking into account that production of phenolic compounds driven by blue light is mediated by cytochrome P450 which leads to ROS (reactive oxygen species) accumulation. As a protective mechanism, ROS scavengers such as rosmarinic and caffeic acids are produced [58]. This is consistent with the higher antioxidant activity recorded in the same treatments where higher phenolic contents were observed, as phenolics are known as potent radical scavengers. Particularly, anthocyanins were shown to improve significantly the antioxidant activity in red compared to green leaves in *Quintinia serrata* [48]. Distinct from the higher accumulation of total phenolics and flavonoids in the red cultivar under predominantly blue illumination, the increased levels of anthocyanins under predominantly red light might be explained by the different regulatory systems of anthocyanins and other polyphenols such as flavonols [59]. However, differences among treatments were not significant and it is known that anthocyanin synthesis is mediated by both red and blue light receptors [60].

The responses of plants to red and blue light in regard to phenolics accumulation is dependent on various factors, among them most important are the species and the cultivation conditions. As such, the highest antioxidant activity and phenolic contents were recorded under predominantly red light in *Valerianella locusta* [22], antioxidant activity decreased with higher red ratios in coriander [31] while highest phenolic contents in tomato stems and leaves were recorded under blue light [61]. In green basil, total phenolics increased under increasing blue light ratios [25], under UV-A [24] or under red light [21]. Caffeic and rosmarinic acid are known to accumulate more in *Perilla frutescens* under artificial light composed 80% red and 20% blue, followed by UV-A lighting compared to natural light contained 13.5% UV-A and 23.5% blue [58]. In green basil, accumulation of total phenolics and rosmarinic acid was highest with white light, followed by red and blue light [62]. In another paper, also in green basil, prolonged blue lighting led to significant accumulation of only cichoric acid and quercetine, but not rosmarinic acid [63]. In the respective papers, basil plants were analyzed at different cultivation periods than in the present study, and had slightly different cultivation conditions, which may account for differences in results.

Microgreens can constitute a valuable source of health maintaining compounds, such as phenolics, enzymes or vitamins and minerals [2]. Improving their qualities is an exciting avenue of research and biotechnology, as properties of microgreens can be enhanced through various stimuli which may be synergic, while also easily available. Improvement of microgreens quality using LEDs has already been described in numerous species, such as rice [27], buckwheat [23], amaranth, pea, kale, broccoli, mustard, basil, borage, beet, parsley [25], basil [64], *Brassica* spp. [19], etc. Rosmarinic acid, a predominant phenolic in *Ocimum basilicum*, has antiviral, antibacterial, anti-inflammatory and antioxidant activities [65], while caffeic acid, also present in basil, has potent antioxidant capacity, due to metal chelating activity and prevention of α-tocopherol consumption and (low density lipoproteins) LDL oxidation [66]. Several possible bioactivities of anthocyanins are relief of oxidative stress, prevention of cardiovascular diseases by antioxidative capacity, anti-inflammatory properties, control of diabetes, improvement of eye vision and antimicrobial activity [67]. As a result, basil microgreens can significantly improve health promoting properties of food when consumed, especially raw.

Considering that both red and blue light influence growth and synthesis of beneficial compounds in microgreens by overlapping mechanisms and that the responses are species or cultivar specific, tailoring the yield and quality of such food becomes a matter of finding the optimal ratios of different light wavelengths and other cultivation conditions.

## 4. Materials and Methods

### 4.1. Plant Material and Growth Conditions

**Figure 7:**
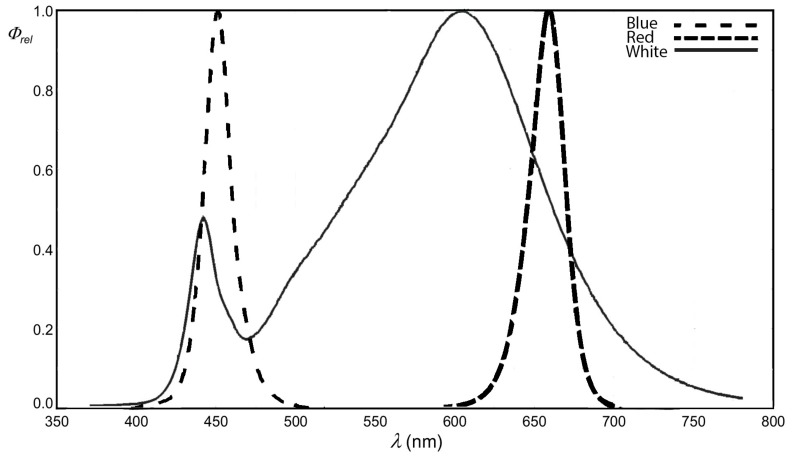
LED emission spectra.

In the current experiment, two basil cultivars were used for microgreens production, “Sweet Genovese”, a green, acyanic cultivar, and “Red Rubin”, a red pigmented, cyanic cultivar, seeds being obtained from a commercial source (Seedaholic Ltd., Clonbur, UK). For each treatment, 150 seeds were sowed in clear plastic boxes (5 boxes per treatment, 3 used for biochemical analyses and 2 for phenotypic measurements), 14 × 14 × 8 cm, using a mixture of general purpose soil and peat moss 2:1. Each light treatment was provided by 6 (2 × 3) 1 W LEDs (OSRAM, Golden Dragon, Munich, Germany), soldered on aluminium radiators to allow heat dissipation. The 4 light treatments were 100% white (White) and various red (R) to blue (B) ratios, as follows: 2R:1B, 1R:1B and 1R:2B, intensities according to Table 4. After seeding, the boxes were kept in the dark for 3 days and afterwards light was supplied at a rate of 120 µmol m^−2^ s^−1^ for 12 h each day. The emission spectra of LED lights (according to OSRAM datasheets) used are given in Figure 7.

### 4.2. Analyses

Total cotyledon area was measured using a Scanalyzer PL (LemnaTec, Aachen, Germany), which allowed photographing the boxes containing microgreens from a fixed height and calculations of areas. Measurements were performed over an 8 days period starting with day 6 after sowing, up to the point where leaves from different individuals began to overlap, preventing accurate measurements. Boxes were photographed sequentially, minimizing the time spent outside the defined light treatment, generally 1 min. The software setup for the phenotyping system was similar to that previously described by Arvidsson et al. [68].

Chlorophyll and carotenoid pigments were extracted in 80% aqueous acetone and quantified by spectrophotometric means using formulas described in Wellburn [69]. Chlorophyll fluorescence was measured using a FMS2 fluorometer (HansaTech, Norfolk, UK) for 10 cotyledons/treatment. Chlorophyll related analyses were performed at the end of the experiment, at 17 days after sowing.

For total phenolics, flavonoids, free radical scavenging activity and anthocyanin evaluations, 10 plantlets were pooled from each of 2 boxes per treatment at defined days, resulting a total of 20 plantlets/treatment/day of assessment. Total phenolics, flavonoids and free radical scavenging activity were assayed in 70% ethanolic extracts, obtained by maceration for 24 h at room temperature, according to methods described in Herald et al. [70]. Anthocyanin determinations were performed according to Lee et al. [71] in methanol:water:hydrochloric acid (70:29:1) mixture extracts. For each assessment, 3 technical replicates were performed.

Individual phenolic acids at 17 days after sowing were determined using a Shimadzu High Performance liquid chromatography (HPLC) (Shimadzu LC-10ADVP, Columbia, MD, USA) coupled to a photodiode array (PDA) detector (Shimadzu SPD-M20A, Columbia, MD, USA). The chromatographic conditions were represented by a Macherey-Nagel C18 reverse phase (150 mm × 4.6 mm × 4 µm) column, water:acetic acid (99:1) (A) and methanol (B) mobile phases, 0.6 mL/min flow rate and 40 °C. The chromatographic program was 0 min 100% A, 5 min 6% B, 5–7 min 6% B, 50 min 30% B, 50–52 min 30% B, 62 min 100% B. Compounds (rosmarinic and caffeic acids) were quantified using external standards, HPLC grade (Sigma, Steinheim, Germany).

## 5. Conclusions

Blue and red light LED treatments have a significant potential of improving growth of basil microgreens while increasing also the contents of phenolic compounds as valuable phytochemicals. Considering the developing interest in microgreens as functional foods, improved quality basil microgreens can be achieved using either predominantly red or blue light, depending on the pigmentation of the cultivar.

## Figures and Tables

**Table 1:** Fresh and dry mass of green basil cultivar.

Treatment	White	2R:1B	1R:1B	1R:2B
Fresh mass (g/100 microgreens)	4.27 ± 0.3 ^ab^	3.67 ± 0.29 ^a^	3.97 ± 0.34 ^a^	5.51 ± 0.31 ^b^
Dry mass (g/100 microgreens)	0.4 ± 0.05 ^ab^	0.34 ± 0.03 ^a^	0.39 ± 0.02 ^ab^	0.58 ± 0.06 ^b^

Results are means ± standard error. Means with the same lowercase letter are not significantly different at *p* < 0.05 according to Tukey’s test.

**Table 2:** Chlorophyll and carotenoid pigment contents in green and red basil cultivars.

Pigment	Cultivar/Treatment	White	2R:1B	1R:1B	1R:2B
Chlorophyll a mg g^−1^	Green cv.	0.5 ± 0.06 ^a^	0.48 ± 0.07 ^a^	0.53 ± 0.04 ^a^	0.68 ± 0.03 ^a^
	Red cv.	0.35 ± 0.01 ^a^	0.39 ± 0.05 ^a^	0.42 ± 0.06 ^a^	0.4 ± 0.04 ^a^
Chlorophyll b mg g^−1^	Green cv.	0.27 ± 0.06 ^a^	0.21 ± 0.03 ^a^	0.33 ± 0.1 ^a^	0.31 ± 0.01 ^a^
	Red cv.	0.24 ± 0.06 ^a^	0.27 ± 0.05 ^a^	0.29 ± 0.06 ^a^	0.3 ± 0.03 ^a^
Carotenoids mg g^−1^	Green cv.	0.1 ± 0.02 ^a^	0.1 ± 0.01 ^a^	0.11 ± 0.01 ^a^	0.15 ± 0.01 ^a^
	Red cv.	0.11 ± 0.02 ^a^	0.1 ± 0.04 ^a^	0.09 ± 0.01 ^a^	0.1 ± 0.03 ^a^

Results are means ± standard error. Means with the same lowercase letter are not significantly different at *p* < 0.05 according to Tukey’s test.

**Table 3:** Selected phenolic acids and anthocyanins contents of red basil cultivar.

Treatment	White	2R:1B	1R:1B	1R:2B
Caffeic acid (mg/g f.w.)	0.62 ± 0.018 ^a^	0.73 ± 0.018 ^b^	1.37 ± 0.015 ^c^	2.57 ± 0.016 ^d^
Rosmarinic acid (mg/g f.w.)	0.33 ± 0.021 ^a^	0.84 ± 0.018 ^b^	1.65 ± 0.034 ^c^	4.99 ± 0.035 ^d^
Anthocyanins (mg/g f.w.)	1.44 ± 0.04 ^a^	2.45 ± 0.05 ^c^	2.19 ± 0.04 ^b^	2.24 ± 0 ^b^

Results are means ± standard error. Means with the same lowercase letter are not significantly different at *p* < 0.05 according to Tukey’s test.

**Table 4:** Light treatment intensities.

Treatment (μmol m^−2^ s^−1^)	White	2R:1B	1R:1B	1R:2B
White	120	-	-	-
Red	-	80	60	40
Blue	-	40	60	80
